# First-in-Human Clinical Evaluation of a Novel Nonsurgical Suprachoroidal Delivery Approach for Triamcinolone in Diabetic Macular Edema

**DOI:** 10.1016/j.xops.2026.101185

**Published:** 2026-04-02

**Authors:** Yoreh Barak, Alexey Rapoport, Keren Mano-Tamir, Anastasiia Adakhovska, Quan Dong Nguyen

**Affiliations:** 1 Department of Ophthalmology, Rambam Health Care Campus, Haifa, Israel; 2 Rappaport Faculty of Medicine, Technion-Israel Institute of Technology, Haifa, Israel; 3 Everads Therapy Ltd, Tel Aviv, Israel; 4 Consultant to Everads Therapy Ltd, Tel Aviv, Israel; 5 Byers Eye Institute, Stanford University, Palo Alto, California

**Keywords:** Diabetic macular edema, Posterior segment therapy, Retinal drug delivery, Suprachoroidal injection, Triamcinolone acetonide

## Abstract

**Purpose:**

To evaluate the safety, tolerability, and effectiveness of the Everads Injector, a novel suprachoroidal drug delivery device, for administration of triamcinolone acetonide (TA) in patients with diabetic macular edema (DME).

**Design:**

A prospective, open-label pilot study.

**Subjects:**

Ten adult patients (10 eyes) with center-involved DME and inadequate response to prior anti-VEGF therapy.

**Methods:**

Each participant received a single suprachoroidal injection of 4 mg of TA in 100 μL using the injector. The device enables rapid, tangential delivery into the suprachoroidal space (SCS) by creating a channel from the sclera into the choroid via blunt dissection. Follow-up assessments were conducted on injection day and on days 3, 14, 28, and 42. Safety was assessed by adverse events monitoring, intraocular pressure (IOP) measurements, and comprehensive ocular examinations. Suprachoroidal delivery was confirmed through clinical evaluation and thermal imaging, verifying successful localization of TA within the SCS. Efficacy was measured by changes in central macular thickness and best-corrected visual acuity (BCVA).

**Main Outcome Measures:**

Safety, device performance, central macular thickness, and BCVA.

**Results:**

Triamcinolone acetonide was successfully delivered into the SCS in all participants, with no serious adverse events reported. Over 5 to 7 seconds, 100 μL of TA was injected. Mild subconjunctival hemorrhages occurred in 7 eyes and resolved without intervention IOP remained stable within the normal physiological range (12–18 mmHg) throughout all follow-up visits. Statistically significant reductions in central macular thickness were observed by day 42 (mean change: –134.9 μm; *P* = 0.0039). Best-corrected visual acuity improved in most participants (8 eyes), with a mean gain of +11.4 letters on the ETDRS chart from baseline to visit 6.

**Conclusions:**

Suprachoroidal delivery of TA in patients with DME using the injector demonstrated favorable safety and tolerability. The procedure was well tolerated under topical anesthesia and performed successfully in an office-based setting. These findings support the feasibility of this delivery technology and highlight its potential clinical benefit in reducing macular edema and improving visual function. Further studies are warranted to confirm these results in larger, controlled trials.

**Financial Disclosure(s):**

Proprietary or commercial disclosure may be found in the Footnotes and Disclosures at the end of this article.

Diabetic macular edema (DME) is the leading cause of vision impairment among individuals with diabetic retinopathy, affecting millions of patients globally and an estimated 7% of diabetic adults over the age of 40 in the United States.^1^^,^^2^ The growing global prevalence of diabetes is expected to increase the burden of diabetic retinopathy and DME significantly over the coming decades.^3^ Diabetic macular edema is characterized by fluid accumulation and retinal thickening due to the breakdown of the blood–retinal barrier, primarily driven by hyperglycemia-induced oxidative stress, ischemia, chronic inflammation, and VEGF overexpression.^1^

Current standard-of-care for center-involved DME primarily relies on intravitreal anti-VEGF agents such as aflibercept, ranibizumab, and bevacizumab, as well as faricimab (combination of anti-VEGF and anti-angiopoietin). These biologics effectively target VEGF-mediated vascular permeability and neovascularization, improving visual acuity and reducing retinal thickness, as demonstrated in several large-scale randomized clinical trials.^4^, ^5^, ^6^ However, routine clinical practice treatment outcomes often fall short of clinical trial benchmarks due to undertreatment, adherence challenges, and variability in disease phenotype.^7^ Corticosteroids offer an alternative or adjunctive therapeutic strategy, especially for DME cases with a strong inflammatory component or poor response to anti-VEGF agents. Sustained-release intravitreal corticosteroid implants have shown efficacy in reducing macular edema and improving vision.^8^ However, their use is often limited by risks of elevated intraocular pressure (IOP) and cataract formation due to anterior-segment drug exposure.^9^^,^^10^

The suprachoroidal space (SCS) has gained traction as a promising route for targeted drug delivery to the posterior segment of the eye. By compartmentalizing therapeutic agents away from anterior-segment structures, SCS delivery allows for enhanced localization of corticosteroids to the retina and choroid while minimizing exposure-related complications. Preclinical pharmacokinetic studies have shown that suprachoroidal triamcinolone acetonide (TA) achieves robust tissue concentrations in the posterior segment with limited diffusion to the anterior chamber.^11^^,^^12^ These findings support the growing interest in SCS delivery for posterior segment diseases and have laid the groundwork for clinical evaluation.

Suprachoroidal delivery of TA for uveitic macular edema has been approved by the U.S. Food and Drug Administration for clinical use in the United States as Xipere (Bausch & Lomb Americas Inc.), following the PEACHTREE and other studies demonstrating its safety and efficacy.^13^

Additionally, as interests in gene therapy for retinal disorders grow, the SCS has emerged as a promising, less invasive route for delivering genetic payloads to the posterior segment.^14^ Gene therapy offers the potential for long-term, 1-time treatment through targeted genetic correction. Voretigene neparvovec-rzyl (Luxturna, Spark Therapeutics), the first Food and Drug Administration and the European Medicines Agency-approved gene therapy for inherited retinal dystrophies caused by biallelic RPE65 mutations, demonstrated successful subretinal delivery of therapeutic genes using adeno-associated viral vectors. However, the procedure requires surgical subretinal injection in an operating room, which carries procedural risks including retinal damage and inflammation. Moreover, distribution of the therapeutic vector can be uneven and localized, making consistent treatment coverage a challenge.^15^

While effective in certain monogenic conditions, broader application of gene therapy in multifactorial diseases like DME remains investigational and faces both technical and anatomical delivery constraints.

The Everads Injector (Everads Therapy Ltd) represents a new generation of suprachoroidal delivery technologies designed for rapid and broad drug distribution throughout the SCS via a nonsurgical, office-based administration. It offers precise depth control and safe delivery of injectate into the SCS, potentially enhancing efficacy and safety while reducing procedural barriers. Unlike the perpendicular microneedle-based suprachoroidal delivery approach, which enters the SCS perpendicularly, the injector enables a tangential injection into the SCS and rapid distribution throughout the SCS by creating a channel between the scleral and choroidal tissues via which therapeutic agents are injected. Preclinical evaluation of the injector in nonhuman primates demonstrated its capacity to enable safe, well-tolerated suprachoroidal delivery with broad and consistent posterior segment distribution, including macular coverage, and no substantial ocular adverse effects.^16^

In this first-in-human, prospective open-label pilot study, we evaluated the safety, tolerability, and preliminary effectiveness of the injector for delivering a single 4-mg dose of TA delivered suprachoroidally using the injector in patients with persistent center-involving DME despite prior anti-VEGF therapy. The open-labeled trial provides critical early clinical data for this novel suprachoroidal delivery technology with the potential to transform posterior segment drug delivery by offering targeted treatment in a minimally invasive format.

## Methods

### Study Design and Oversight

The index trial was a single-center, open-label, prospective pilot study, designed to evaluate the safety and performance of suprachoroidal delivery of TA using the injector in patients with DME. This study was prospectively registered at ClinicalTrials.gov (Identifier: NCT06314217). The study enrolled 10 adult subjects (8 males and 2 females) who met all eligibility criteria. Each participant received a single 4-mg (100 μL) dose of TA administered into the SCS.

Institutional review board/ethics committee approval was obtained.

The study was approved by the Ethics Committee of Rambam Health Care Campus, Haifa, Israel, in addition to approval by the Israeli Ministry of Health.

All subjects provided written informed consent prior to participation.

The study was conducted in accordance with the Declaration of Helsinki, International Conference on Harmonisation Good Clinical Practice, International Organization for Standardization 14155, and relevant national regulations.

Per request of the Israeli Ministry of Health, initial enrollment was restricted to 3 patients, each with an ETDRS best-corrected visual acuity (BCVA) letter score ≤35 (Snellen equivalent 20/200) in the study eye and ≥60 (20/63) in the fellow eye. Following study completion of these initial patients and submission of an interim safety report, the Israeli Ministry of Health authorized continuation. Subsequently, 7 additional patients were enrolled with a slightly less restrictive visual acuity threshold (study eye ETDRS BCVA ≤50, Snellen equivalent 20/100).

### Patient Population

Participants were eligible for inclusion in the study if they were 18 years of age or older and had a confirmed diagnosis of type 1 or type 2 diabetes mellitus with center-involving DME. Central involvement was defined by a central subfield thickness of ≥320 μm for males and ≥305 μm for females when measured by Spectralis spectral-domain OCT (SD-OCT), or ≥305 μm for males and ≥290 μm for females when measured by Cirrus SD-OCT. Eligible participants were also required to meet specific visual acuity criteria outlined in the protocol and to have demonstrated an inadequate response to at least 3 prior intravitreal injections of anti-VEGF agents. All participants were required to provide written informed consent and to demonstrate a willingness and ability to comply with study procedures and visit schedules. In addition, women of childbearing potential were required to agree to use effective contraception throughout the duration of the study.

Patients were excluded from participation if they had macular edema attributable to any cause other than diabetic retinopathy or if they had undergone ocular surgery or received laser photocoagulation in the study eye within 90 days prior to screening. Additional exclusion criteria included uncontrolled IOP (defined as IOP ≥ 21 mmHg or the need for more than 2 IOP-lowering medications), prior treatment with corticosteroid implants or intravitreal injections within the defined exclusion windows, and the presence of active intraocular inflammation. Participants were also excluded if they had significant coexisting ocular pathologies that could interfere with study assessments or outcomes, or if they had a known hypersensitivity to TA, anesthetic agents, fluorescein, indocyanine green, or any other study-related substances. Individuals with uncontrolled systemic conditions, a hemoglobin A1c level greater than 12%, or who were pregnant or breastfeeding at the time of screening were also ineligible for enrollment (see Table S1, available at www.ophthalmologyscience.org for full eligibility criteria).

### Investigational Device

**Figure 1:**
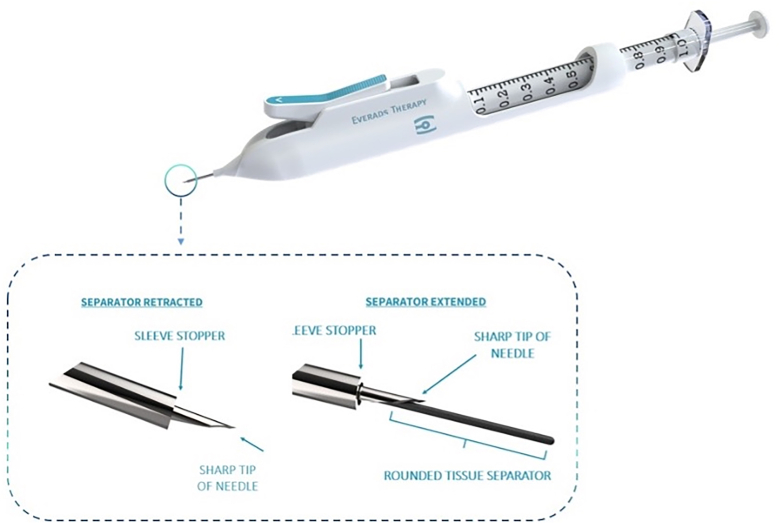
The injector includes a blunt tissue separator shown in both extracted and retracted positions, along with a sleeve stopper that restricts needle depth during tangential scleral entry.

**Figure 2:**
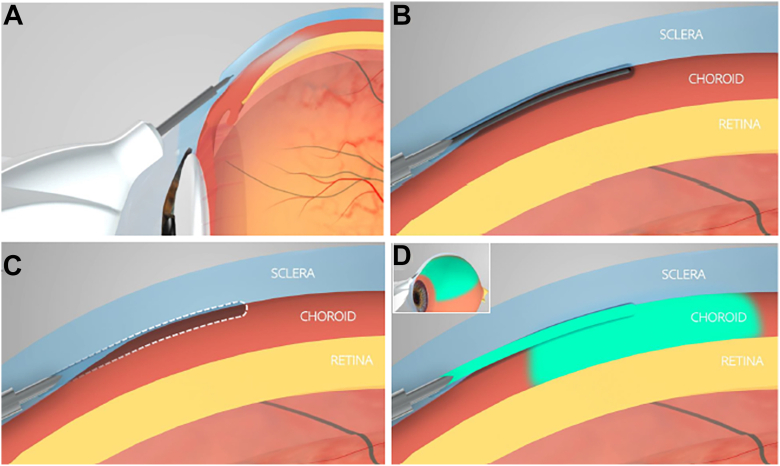
Suprachoroidal delivery using the injector. **A,** Tangential insertion of the injector's bevel into the sclera, with the sleeve stopper controlling entry depth. **B,** Extension of the nonsharp tissue separator to create a path into the SCS. **C,** Retraction of the separator, leaving a channel to the SCS. **D,** Injection and posterior distribution of the therapeutic agent. SCS = suprachoroidal space.

**Figure 3:**
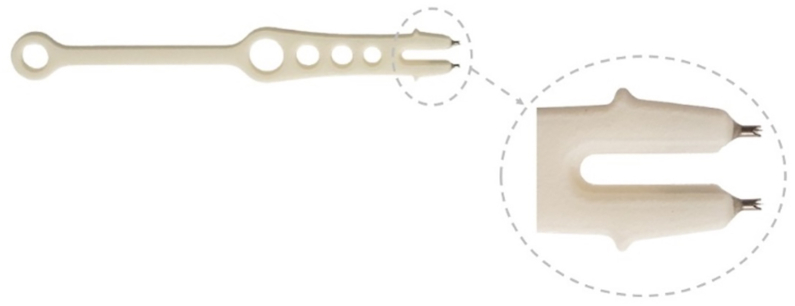
The fixation tool, an optional single-use sterile accessory designed to stabilize the eye during injection. The prongs shown at the tip of the device enable secure engagement of the sclera through the conjunctiva, providing counterforce to support tangential scleral entry.

This study used a sterile, single-use suprachoroidal injection delivery device, engineered for nonsurgical access to the SCS. The device, which is inserted at a tangential angle, incorporates a standard 27-gauge ultra-thin-walled needle housing a retractable nonsharp tissue separator designed for blunt dissection between the sclera and choroid, enabling controlled and nonpenetrative access to the SCS. The injector is designed to work with standard 1-mL Luer-lock tuberculin syringes. A key safety feature of the injector is the sleeve stopper, which restricts the advancement depth of the needle once it is inserted tangentially into the scleral surface (Fig 1). Once the clinician advances the needle tangentially into the sclera, the clinician depresses the actuator to deploy the tissue separator. This action transiently opens the suprachoroidal plane by creating a controlled mechanical separation of tissues. Upon release of the actuator, the separator automatically retracts into the device, following which the injectate is delivered by depressing the plunger of the syringe (Fig 2). To support procedural precision, the fixation tool could be used at the discretion of the principal investigator to facilitate atraumatic globe stabilization by securing the conjunctiva and optimizing scleral access during injection (Fig 3).

### Study Treatment and Administration

In this trial, a single dose of preservative-free TA suspension (Intracinol, Farmigea; 4 mg in 100 μL) was administered to the designated study eye via suprachoroidal injection using the injector. The fellow eye remained untreated within the trial protocol but could receive standard-of-care treatment at the investigator's discretion. All injections were performed under topical anesthesia with oxybuprocaine using aseptic technique. The suprachoroidal injection procedure is shown in Video S1 (available at www.ophthalmologyscience.org).

### Thermal Camera

A handheld infraed thermal camera was employed during the study to monitor ocular surface temperature dynamics throughout the injection procedure. This device detects emitted infrared radiation and translates it into a thermal image, enabling real-time visualization of subtle temperature changes. Thermal imaging was performed continuously during the injection to evaluate the thermal gradient at the injection site. Thermal imaging of the suprachoroidal injection is presented in Video S2 (available at www.ophthalmologyscience.org).

### Follow-Up and Study Visits

Following injection-day assessments, all subjects underwent four follow-up visits to monitor safety, tolerability, and treatment response. These visits occurred on day 3 (±1), day 14 (±3), day 28 (±3, conducted via telephone for safety monitoring), and day 42 (±3), which marked the end-of-study evaluation. Each in-person visit involved a comprehensive evaluation comprising both ocular and systemic assessments. These included BCVA testing, slit lamp biomicroscopy, dilated indirect ophthalmoscopy, and IOP measurement. Multimodal imaging was also performed, including SD-OCT, enhanced depth imaging OCT, fluorescein angiography, indocyanine green angiography, and widefield fundus photography. To ensure consistency and reliability of imaging data, all procedures were performed using the same equipment and by the same trained personnel. The detailed schedule of study procedures and assessments is provided in Table S2 (available at www.ophthalmologyscience.org).

### Outcome Measures

Outcome measurements focused both on evaluating the safety and tolerability of suprachoroidal delivery using the investigational device, and the safety and efficacy of the TA. Safety assessments included continuous monitoring of treatment-emergent adverse events, adverse device effects, and serious adverse events (SAEs), along with IOP measurements and changes in BCVA. These evaluations were conducted during all in-person follow-up visits and were supplemented by detailed ocular examinations, including slit lamp biomicroscopy and dilated indirect ophthalmoscopy.

Ophthalmic imaging and functional testing were integral components of the outcome assessments. Best-corrected visual acuity was measured using standardized ETDRS charts to ensure consistency and comparability across visits. Intraocular pressure was assessed using Goldmann applanation tonometry, the clinical gold standard for IOP measurement, and was taken approximately 30 minutes postinjection. Retinal and choroidal thickness were quantified through SD-OCT and enhanced depth imaging OCT. Fluorescein angiography, indocyanine green angiography, and widefield fundus photography were performed at predefined time points to characterize retinal and choroidal morphology, confirm suprachoroidal drug delivery, and evaluate treatment-related anatomical changes. Successful suprachoroidal delivery also was confirmed by a characteristic cooling pattern consistently observed on thermal imaging. This pattern, extending posterior to the needle entry point, was interpreted as indicative of correct drug placement in the SCS.

### Statistical Analysis

Descriptive statistics were employed for demographic, clinical, and safety data. Categorical data were summarized using frequencies and percentages, while continuous variables were described using means, standard deviations, medians, and 95% confidence intervals. The Wilcoxon signed-rank test was used for evaluating changes from baseline in paired data. Analyses were conducted using SAS version 9.4 (SAS Institute). A predefined Statistical Analysis Plan guided all analyses.

## Results

### Patients

Between February 25, 2024, and January 16, 2025, a total of 14 individuals were screened for study eligibility at a single clinical site. Of these, 10 patients (10 unique eyes) met all inclusion and exclusion criteria, were enrolled, received a single suprachoroidal injection of a TA suspension approved for intraocular use via the injector, and completed the study protocol. Four individuals did not qualify for enrollment: 3 were excluded due to the presence of choroidal neovascularization, and one due to recent ocular surgery less than 90 days prior to screening. Demographic and baseline ocular characteristics of the enrolled cohort are summarized in Table 1.

**Table 1:** Baseline Characteristics of Subjects (N = 10)

Characteristic	Value
Gender	
Male, n (%)	8 (80%)
Female, n (%)	2 (20%)
Age (yrs)	
Mean	68.2
SD	7.3
Median	67.5
Min, Max	56, 82
Race	
White, n (%)	10 (100%)
Asian, n (%)	0
Black or African American, n (%)	0
Other, n (%)	0
Diabetic retinopathy grading	
No DR, n (%)	0
Mild NPDR, n (%)	10 (100%)
Moderate NPDR, n (%)	0
Severe NPDR, n (%)	0
PDR, n (%)	0
ETDRS BCVA score	
Mean	41.4
SD	9.1
Median	42
Min, Max	28, 50
CMT (μm)	
Mean	518.3
SD	202.2
Median	442.5
Min, Max	320, 974
IOP (mmHg)	
Mean	14.6
SD	1.3
Median	15
Min, Max	12, 16
Lens status	
Pseudophakic, n (%)	8 (80%)
Phakic, n (%)	2 (20%)

BCVA = best-corrected visual acuity; CMT = central macular thickness; DR = diabetic retinopathy; IOP = intraocular pressure; max = maximum; min = minimum; NPDR = nonproliferative diabetic retinopathy; PDR = proliferative diabetic retinopathy; SD = standard deviation.

This table summarizes demographic and baseline ocular characteristics of the study population at enrollment. Data are presented as mean ± standard deviation, median (range), or number (percentage).

### Safety Evaluation

#### Adverse Events

All 10 patients (10 eyes) completed the study without any SAEs. The injection procedure was well tolerated across the cohort.

Seven participants (7 eyes) exhibited mild subconjunctival hemorrhages in the treated eye immediately postinjection. These events were classified as adverse device effects, resolved spontaneously, and required no clinical intervention. Importantly, they did not affect the success of drug delivery or treatment outcomes. No treatment-emergent adverse events were attributed to the TA formulation, and there were no systemic or ocular SAEs observed throughout the study. The use of topical anesthetic alone was sufficient for all patients; subconjunctival lidocaine, although permitted by protocol, was not utilized. General health and ocular stability were maintained throughout the follow-up period, with no signs of inflammation, tissue reaction, or injection-related complications on slit lamp biomicroscopy or indirect ophthalmoscopy.

#### Intraocular Pressure

Intraocular pressure remained stable and within physiological limits (12–18 mmHg) at all assessed follow-up time points in all treated eyes.

**Figure 4:**
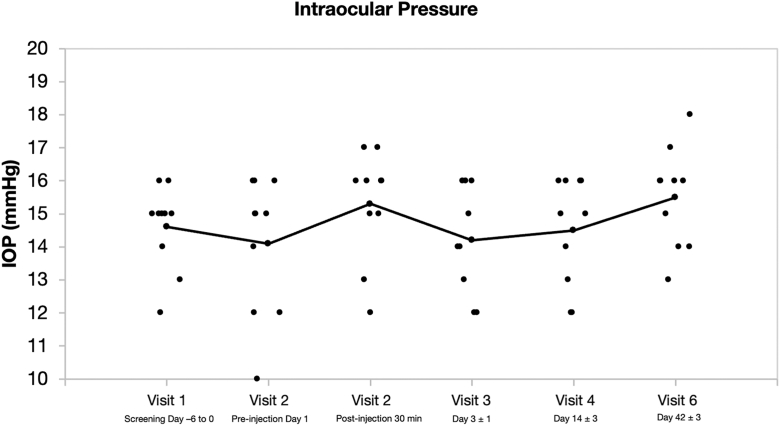
Intraocular pressure measurements by visit/day. IOP = intraocular pressure.

Mean IOP values remained consistent from baseline through the final visit, with individual patient measurements displayed as separate data, and no evidence of acute elevations or clinically significant postinjection fluctuations (Fig 4).

### Device Performance

**Figure 5:**
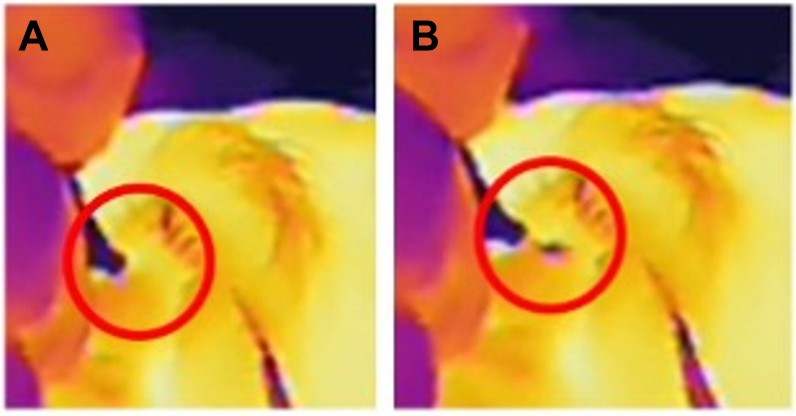
Thermal camera images captured before and during the injection demonstrating the expected posterior flowing SCS injection pattern. **A,** The preinjection thermal image depicts the cooler injector appearing in purple, contrasted against the warmer ocular surface, which is displayed in yellow tones. **B,** The image captured during injection reveals a distinct purple region, visible posterior to the injection site. This area represents localized cooling of the sclera overlying the SCS, corresponding to the entry and dispersion of the TA within the SCS. SCS = suprachoroidal space; TA = triamcinolone acetonide.

Triamcinolone acetonide was successfully delivered into the SCS in all 10 subjects (10 eyes) confirmed by thermal imaging and ocular examination. Thermal imaging captured during the injection procedures revealed a distinct posterior cooling pattern on the ocular surface, consistent with the injector's delivery method and indicative of successful suprachoroidal administration (Fig 5). Thermal imaging was used in all but 1 patient (1 eye), where technical constraints prevented the imaging capture. For all 10 eyes, successful administration was also verified through ophthalmic assessments, which revealed no presence of drug in nontarget compartments such as the vitreous, retina, or sclera.

One subject experienced partial subconjunctival deposition of TA during the initial injection attempt due to abrupt ocular movement resulting in a transient shift in injector angle. Following clarification to the patient regarding the importance of maintaining stable fixation and avoiding ocular movement during injection, a second injection achieved successful suprachoroidal delivery.

No device malfunctions were reported, and the fixation tool was used to stabilize the eye without incidence in selected cases per the discretion of the investigator.

### Efficacy Evaluation

#### Central Macular Thickness

**Figure 6:**
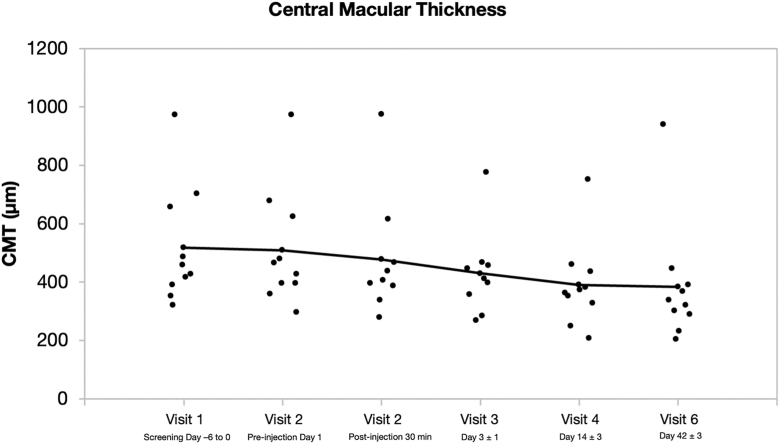
Change in CMT by visit/day. CMT = central macular thickness.

By day 42 (visit 6), a reduction in central macular thickness was observed in most patients (7 eyes). The mean central macular thickness decreased by 134.9 μm (±154.93) from baseline to visit 6, representing a statistically significant change (*P* = 0.0039) (Fig 6). Seven of the 10 subjects exhibited substantial anatomical improvement, while the remaining 3 maintained stable macular thickness throughout the follow-up period. These results were consistently supported by SD-OCT assessments.

#### Best-Corrected Visual Acuity

**Figure 7:**
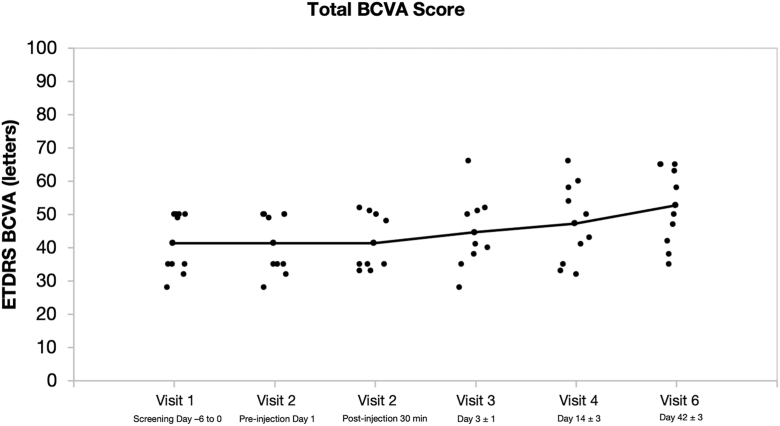
Change in BCVA by visit. BCVA = best-corrected visual acuity.

Mean BCVA, measured by ETDRS letter score, improved from 41.4 at baseline to 52.8 at the end of the study, representing a mean gain of +11.4 letters (±9.01) (Fig 7). Eight eyes demonstrated clinically meaningful improvements in visual function, and no declines in BCVA were recorded during the study period for any study or fellow eyes.

## Discussion

The index study presents the first clinical evidence supporting the feasibility and safety of a novel, nonsurgical suprachoroidal delivery injector for corticosteroid administration in patients with DME. The injector enabled targeted delivery of TA into the SCS under topical anesthesia. Across all 10 patients (10 eyes), the procedure was well tolerated, with no SAEs reported. Successful suprachoroidal drug localization throughout SCS was achieved in all cases. These findings are consistent with a growing body of evidence supporting the suprachoroidal route as a targeted, compartmentalized method for delivering therapeutics to the posterior segment of the eye.^11^^,^^12^

The SCS offers a distinct anatomical advantage by providing direct access to the choroid and retina while minimizing exposure to anterior-segment structures. Such compartmentalization reduces the risk of corticosteroid-associated complications commonly observed with intravitreal administration, such as an increase in IOP. The clinical utility of suprachoroidal corticosteroid delivery has been well illustrated in the PEACHTREE trial, where administration of TA via the microneedle-based suprachoroidal approach led to meaningful improvements in visual acuity and macular thickness in patients with uveitic macular edema. Importantly, the treatment demonstrated a more favorable safety profile compared to intravitreal corticosteroids, with reduced incidences of IOP elevation and cataract progression.^13^^,^^17^ These outcomes reinforce the potential of the suprachoroidal route as a safer and more effective alternative for delivering corticosteroids in inflammatory retinal diseases.

The SCS has garnered increasing attention as a promising route for targeted drug delivery to the posterior segment, offering distinct anatomical and pharmacokinetic advantages. Beyond corticosteroid administration, its potential has expanded into the realm of gene therapy, where localized delivery, immune privilege, and reduced risk of anterior-segment exposure are critical considerations. Recent preclinical investigations have demonstrated the feasibility of delivering adeno-associated viral vectors via the SCS to achieve widespread transgene expression in the choroid, retinal pigment epithelium, and outer retina while minimizing surgical invasiveness and off-target effects. For instance, these studies highlighted the biomechanical advantages of suprachoroidal injection for large-molecule and vector-based therapies, citing favorable vector dispersion and safety in nonhuman primate models.^18^ Additionally, such findings emphasized the growing utility of the SCS for emerging gene-editing platforms and RNA-based therapeutics, suggesting its compatibility with a broad range of viral and nonviral delivery systems.^14^^,^^19^ The established subretinal gene therapy paradigm, while a landmark in subretinal gene therapy, relies on surgical subretinal injection with associated risks and limited vector distribution. In contrast, the SCS enables office-based access to broader retinal areas, potentially improving gene therapy coverage and tolerability. These developments position suprachoroidal administration as a compelling nonsurgical alternative for future retinal gene therapy protocols, particularly for conditions requiring macular or pan-retinal delivery.^15^

Suprachoroidal access can be achieved using different insertion geometries, each associated with distinct technical considerations. Perpendicular microneedle-based systems employ a fixed needle length and require strict 90° alignment, rendering access inherently angle-dependent. Tangential approaches advance along the scleral curvature to establish a controlled sclerochoroidal plane prior to injection and may be less influenced by variations in scleral thickness. Regardless of geometry, injector positioning and stable patient fixation remain essential to ensure consistent suprachoroidal delivery.

The present study provides preliminary clinical evidence from a small cohort suggesting a favorable short-term safety profile for suprachoroidal TA delivery using this approach.

Throughout the 42-day follow-up, IOP remained within physiological limits for all participants, with no sustained elevations or acute spikes. Mild subconjunctival hemorrhages, observed in 7 subjects, were transient and did not impact drug administration or outcomes.

The injector demonstrated consistent performance, with both thermal imaging and clinical assessment confirming accurate suprachoroidal delivery of TA. No cases of misdelivery into the vitreous, retina, or sclera were detected. A single instance of partial subconjunctival deposition was effectively managed, illustrating the operator-controlled and adaptable nature of the device.

Anatomical and functional improvements further support the therapeutic potential of this approach. By day 42, injected eyes exhibited a statistically significant mean reduction in central macular thickness of 134.9 μm (*P* = 0.0039), and a mean gain of +11.4 ETDRS BCVA, exceeding the threshold for clinical significance. These results are consistent with early-phase trials evaluating suprachoroidal corticosteroids in DME and affirm the potential for this route in patients who had suboptimal response to anti-VEGF therapy.^7^ While corticosteroids remain an essential therapeutic option for DME, their intravitreal use is often limited by tolerability concerns. Elevated IOP, cataractogenesis, and floaters have all been associated with direct anterior-segment exposure following intravitreal administration.^20^ Suprachoroidal delivery offers an anatomical and pharmacological advantage by selectively targeting posterior tissues while sparing anterior structures, potentially improving both efficacy and safety in this patient population.

The current study has inherent limitations, including its small sample size, short duration of follow-up, and absence of a control group. Nonetheless, the favorable safety profile, targeted delivery, and encouraging anatomical and visual outcomes provide important early evidence supporting the clinical utility of suprachoroidal TA delivery using the injector.

Looking ahead, larger randomized controlled trials with longer-term follow-up are warranted to confirm these preliminary findings and to evaluate durability, comparative efficacy, and potential use in combination strategies. The SCS also presents a promising conduit for emerging gene therapies aimed at the posterior segment, offering enhanced precision and reduced procedural burden.^19^

In conclusion, this innovative approach represents a significant advancement in posterior-segment drug delivery, utilizing the anatomical advantages of the SCS to enable targeted, macula-focused therapy while limiting exposure to anterior-segment structures. Building on preclinical evidence in nonhuman primates, the present findings highlight the clinical translatability of the injector for the treatment of DME and potentially other posterior-segment diseases, including other retinal vascular diseases as well as inherited retinal diseases in clinical practice settings.
