# Supplementary material for: First-in-Human Clinical Evaluation of a Novel Nonsurgical Suprachoroidal Delivery Approach for Triamcinolone in Diabetic Macular Edema
Source: Ophthalmol Sci. 2026 Apr 2;6(6):101185. doi: 10.1016/j.xops.2026.101185 (PMC13199977; doi:10.1016/j.xops.2026.101185)
Supplement: Table S1 [file mmc1.pdf]

Supplementary Table 1. Study Eligibility Criteria

This table lists the full inclusion and exclusion criteria used to determine eligibility for study enrollment.

Inclusion Criteria

1. Males and females ≥ 18 years of age
2. Diagnosis of type 1 or type 2 diabetes mellitusκa
3. Diagnosis of DME with confirmed central involvement of ≥ 320 μm for males and ≥ 305 μm for females on Spectralis (Heidelberg) or ≥ 305 μm for males and ≥ 290 μm for females with Cirrus (Zeiss) by spectral domain optical coherence tomography (SD-OCT), verified at the screening visit.
4. First three patients: ETDRS BCVA letter score of worse than 35 (Snellen equivalent of 20/200 imperial or 6/60 metric) in the study eye, with fellow eye ETDRS BCVA of 60 (Snellen equivalent of 20/63 imperial or 6/19 metric) or better.
   Remaining patients: ETDRS BCVA letter score of worse than 50 (Snellen equivalent of 20/100 imperial or 6/30 metric) in the study eye, with fellow eye ETDRS BCVA of 60 (Snellen equivalent of 20/63 imperial or 6/19 metric) or better (rest of patients).
5. Have shown no response to three previous IVT treatments with anti-VEGF agents.
6. Understands the language of the informed consent; willing and able to provide written informed consent prior to any study procedures; willing to comply with the instructions and attend all scheduled study visits.
7. For subject at childbearing age: agreement to remain abstinent or use acceptable contraceptive methods during the study.

Exclusion Criteria

1. Evidence of macular edema due of any cause other than diabetic retinopathy in the study eye.
2. Prior treatment with pan-retinal photocoagulation or focal laser photocoagulation in the study eye within 90 days of screening visit.

3. Intraocular pressure ≥ 21 mmHg or uncontrolled glaucoma (open-angle or angle-closure) in the study eye, requiring more than two antiglaucoma medications.
4. History of any prior ophthalmic surgeries in the study eye within 90 days of the screening visit.
5. Patients for whom enrollment into the study, in the PI's opinion, would put the study eye at undue risk for vision loss.
6. Previous treatment for DME in the study eye with any intravitreal injection in the 30 days prior to the screening visit.
7. Any previous treatment with Ozurdex® implant in past 6 months.
8. Any previous treatment with Iluvien® or Retisert™.
9. Currently enrolled in an investigational drug or device study or has used an investigational drug or device within 30 days of the Screening visit.
10. Any other pre-existing eye conditions or surgical complications that would interfere with the interpretation of study endpoints.
11. Current iris neovascularization, vitreous hemorrhage, tractional retinal detachment or epiretinal membrane that contributes to macular edema in the affected eye, per PI discretion.
12. Known hypersensitivity to any component of or to any of the substances used during the study, including but not limited to TA, anesthetics, fluorescein, and ICG.
13. Known allergy to nickel
14. Uncontrolled blood pressure
15. HbA1c > 12%
16. Pregnant or breastfeeding.

Abbreviations:

BCVA – Best-Corrected Visual Acuity
DME – Diabetic Macular Edema
ETDRS – Early Treatment Diabetic Retinopathy Study
ICG – Indocyanine Green
IVT – Intravitreal
PI – Principal Investigator

SD-OCT – Spectral-Domain Optical Coherence Tomography

TA – Triamcinolone Acetonide

VEGF – Vascular Endothelial Growth Factor

HbA1c – Hemoglobin A1c
