# Supplementary material for: First-in-Human Clinical Evaluation of a Novel Nonsurgical Suprachoroidal Delivery Approach for Triamcinolone in Diabetic Macular Edema
Source: Ophthalmol Sci. 2026 Apr 2;6(6):101185. doi: 10.1016/j.xops.2026.101185 (PMC13199977; doi:10.1016/j.xops.2026.101185)
Supplement: Table S2 [file mmc2.pdf]

Supplementary Table 2. Study Schedule of Events

This table summarizes all protocol-required assessments and procedures at each study visit.

	**Screening Day -6 to 0**	**Baseline Day 1**	**Follow up Day 3 ±1**	**Follow up Day 14 ±3**	**Follow up Day 28 ±3**	**End of Trial Day 42 ±3**
**Visit**	**1**	**2**	**3**	**4**	**5[12]**	**6**
Informed Consent[1]	X					
Demographics[2]	X					
Medical/Ophthalmic History	X					
Physical Examination and Vitals[3, 4]	X	X	X	X		X
Pregnancy Test[5]	X					
Inclusion/ Exclusion	X	X				
HbA1c	X					
Concomitant Medications	X	X	X	X	X	X
Adverse Events		X	X	X	X	X
ETDRS BCVA	X	X[6, 9]	X	X		X
Intraocular Pressure	X	X[6, 8]	X	X		X
EDI-OCT	X	X[6, 7, 10]	X	X		X
SD-OCT	X	X[6, 9]	X	X		X
Fundus Photography[11]	X	X[6, 9]	X			X
Fluorescein Angiography	X					X
ICG Angiography	X		X			X
Indirect Ophthalmoscopy/ Slit Lamp Biomicroscopy	X	X[6, 7, 9]	X	X		X
TA administration by Study Device[13]		X				

Notes

[1] Written informed consent must be obtained prior to conduct of any study-related procedure
[2] Demographics include date of birth, age, sex, race, height, weight, body mass index (BMI)
[3] Physical examination includes: general examination, head, eyes, ears, nose, throat, nails, skin, teeth, tongue, heart, lungs, abdomen, extremities and musculoskeletal and central nervous system assessment (pupils, motor, sensory systems and reflexes)
[4] Vital signs include: body temperature, pulse rate and blood pressure after resting for 5 minutes
[5] Urine pregnancy test: for females at childbearing age
[6] Visit 2 Baseline - Ophthalmological examinations to be performed at baseline (pre-injection): Intraocular pressure (IOP), ETDRS BCVA, SD-OCT, EDI-OCT, fundus photography, slit-lamp biomicroscopy, dilated indirect ophthalmoscopy
[7] Visit 2 immediately after injection – indirect ophthalmology and EDI-OCT
[8] Intraocular pressure to be measured 30±10 minutes post-injection. If IOP remains elevated (≥ 30 mmHg), subject must remain on site until IOP is under control per investigator judgement. If IOP is < 30 mmHg, the subject may leave the clinic after completion of post injection examinations.
[9] Visit 2 postinjection - up to 1h post injection: ETDRS BCVA, SD-OCT, Fundus photography, Slit-lamp biomicroscopy.
[10] Visit 2 post-injection - Additional EDI-OCT might be performed, as per the PI’s discretion 2-3h post-injection
[11] When both fundus photos and FA are conducted in the same visit, the fundus photos should be taken first
[12] Visit 5 will be conducted by phone call to the subject
[13] Thermal imaging may be used to during injection to assess injection into the SCS

Abbreviations:

BCVA – Best-Corrected Visual Acuity

BMI – Body Mass Index

EDI-OCT – Enhanced Depth Imaging Optical Coherence Tomography

ETDRS – Early Treatment Diabetic Retinopathy Study

FA – Fluorescein Angiography

ICG – Indocyanine Green

IOP – Intraocular Pressure

OCT – Optical Coherence Tomography

PI – Principal Investigator

SD-OCT – Spectral-Domain Optical Coherence Tomography

SCS – Suprachoroidal Space

TA – Triamcinolone Acetonide
